# Polygenic association of glomerular filtration rate decline in world trade center responders

**DOI:** 10.1186/s12882-022-02967-5

**Published:** 2022-10-28

**Authors:** Farrukh M. Koraishy, Frank D. Mann, Monika A. Waszczuk, Pei-Fen Kuan, Katherine Jonas, Xiaohua Yang, Anna Docherty, Andrey Shabalin, Sean Clouston, Roman Kotov, Benjamin Luft

**Affiliations:** 1 Division of Nephrology, Department of Medicine, Stony Brook University, 100 Nicolls Road, HSCT16-080E Stony Brook, NY USA; 2 Department of Family, Population, and Preventative Medicine, Stony Brook University, Stony Brook, NY USA; 3 Department of Psychology, Rosalind Franklin University, North Chicago, IL USA; 4 Department of Applied Mathematics and Statistics, Stony Brook University, Stony Brook, NY USA; 5 Department of Psychiatry, Stony Brook University, Stony Brook, NY USA; 6 Department of Medicine, Stony Brook University, Stony Brook, NY USA; 7 Department of Psychiatry, University of Utah, Salt Lake City, UT USA

**Keywords:** Estimated glomerular filtration rate (eGFR), Chronic kidney disease (CKD), Polygenic risk score (PRS)

## Abstract

**Background:**

The factors associated with estimated glomerular filtrate rate (eGFR) decline in low risk adults remain relatively unknown. We hypothesized that a polygenic risk score (PRS) will be associated with eGFR decline.

**Methods:**

We analyzed genetic data from 1,601 adult participants with European ancestry in the World Trade Center Health Program (baseline age 49.68 ± 8.79 years, 93% male, 23% hypertensive, 7% diabetic and 1% with cardiovascular disease) with ≥ three serial measures of serum creatinine. PRSs were calculated from an aggregation of single nucleotide polymorphisms (SNPs) from a recent, large-scale genome-wide association study (GWAS) of rapid eGFR decline. Generalized linear models were used to evaluate the association of PRS with renal outcomes: baseline eGFR and CKD stage, rate of change in eGFR, stable *versus* declining eGFR over a 3–5-year observation period. eGFR decline was defined in separate analyses as “clinical” (> -1.0 ml/min/1.73 m^2^/year) or “empirical” (lower most quartile of eGFR slopes).

**Results:**

The mean baseline eGFR was ~ 86 ml/min/1.73 m^2^. Subjects with decline in eGFR were more likely to be diabetic. PRS was significantly associated with lower baseline eGFR (*B* = -0.96, *p* = 0.002), higher CKD stage (*OR* = 1.17, *p* = 0.010), decline in eGFR (*OR* = 1.14, *p* = 0.036) relative to stable eGFR, and the lower quartile of eGFR slopes (*OR* = 1.21, *p* = 0.008), after adjusting for established risk factors for CKD.

**Conclusion:**

Common genetic variants are associated with eGFR decline in middle-aged adults with relatively low comorbidity burdens.

**Supplementary Information:**

The online version contains supplementary material available at 10.1186/s12882-022-02967-5.

## Introduction

The overall prevalence of chronic kidney disease (CKD) is ~ 15% among adults in the United States [1]. The prevalence is comparatively lower in younger individuals (~ 6%, ~ 12% and ~ 38% for individuals in age groups 18–44, 45–64 and over 65 years receptively) [1]. A decline in glomerular filtration rate (GFR) has been associated with progression of CKD [2]. CKD is associated with significant healthcare costs, as well as morbidity and mortality, [3] and recent data suggest that even mild reductions in estimated (e)GFR is associated with adverse long-term outcomes [4–6]. While eGFR is measured routinely in clinical practice, urine albumin measurements are not performed as frequently [7], and individuals with eGFR > 60 ml/min/1.73 m [2] who develop early decline in kidney function often remain undiagnosed [6]. Consequently, in observational studies using electronic health record (EHR) data, a significant decline in eGFR might be a better measure of early kidney disease than a clinical diagnosis of ‘incident CKD’ [8] that requires albuminuria assessment.

Identifying a patient at risk for GFR decline at an early stage might provide better opportunities for focused preventive therapies. Well-established risk factors such as demographics (age, gender, & race/ethnicity) and clinical comorbidities (e.g. diabetes mellitus, hypertension, and cardiovascular disease) only partially explain overall risk for CKD [9]. There are only a few published studies that report predictors and correlates of early eGFR decline in low risk adults (young and middle-aged adults with a low comorbidity burden i.e. low prevalence of diabetes mellitus, hypertension, and cardiovascular disease) [6, 10]. Therefore, researchers and clinicians might benefit from incorporating new methods to improve risk assessment.

As shown in biometric analyses of twin and family data [11–15], as well as molecular genetic studies [16–21], CKD tends to cluster within families. GFR decline is a heritable trait (h^2^ = 0.36–0.75) [22]. Genome-wide association studies (GWAS) have identified numerous risk loci associated with kidney dysfunction [17, 18]. While most previous GWAS that evaluated renal phenotypes utilized cross-sectional eGFR [17, 23−25] or annualized slope of decline in eGFR [20, 26, 27], Gorski et al. [19] recently published the largest meta-analysis of rapid eGFR decline to date, that included GWAS data from 42 longitudinal studies with findings from > 270,000 individuals. While these GWAS provide new insights into the complex etiology and pathogenesis of CKD [23], direct clinical application of this information has been hindered by the fact that most individual polymorphisms explain only a very small proportion of risk. The limited predictive potency of individual polymorphisms has led to the development of polygenic risk scores (PRS), which aggregate the estimated effects of numerous SNPs into a single genetic risk score.

PRSs have been featured in previous studies to determine the risk of kidney disease. [23, 28−31]. For example, Fujii et al. [31] developed a PRS by combining 18 significant SNPs and reported an adjusted odds ratio (aOR) of 1.12 (1.04, 1.20) of having CKD. Likewise, Yun et al. used 12 significant SNPs to calculate a PRS that was significantly associated with incident CKD (HR 1.31, 95% C.I. = 1.20–1.43). Ma et al. [28], developed a PRS using 53 SNPs associated with eGFR in a large GWAS study [32] of participants in the Framingham cohort, and observed an OR of incident Stage III CKD of 1.37 (1.02–1.83). Hellwege et al. [23] developed a PRS of eGFR using the GWAS summary statistics from the CKDGen Consortium [21, 32]. Finally, Thio et al. [29] constructed a PRS that was associated with baseline GFR and CKD using 53 significant SNPs from the GWAS by Pattaro et al. [32]. To date, few studies have attempted to test the utility of PRSs to predict early GFR decline in independent populations of young and middle-aged adults.

To address these gaps in literature, we explored the association of a PRS generated from the aforementioned GWAS meta-analysis [19] with multiple renal outcomes (based on baseline and longitudinal measurements of eGFR) among responders enrolled in the World Trade Center (WTC) Health and Wellness Program [33]. Because eGFRs are not routinely measured in middle-aged relatively healthy adults, data available from the WTC cohort provides a unique opportunity to assess the predictive value of PRS for eGFR decline prior the emergence of advanced CKD. Additionally, the WTC first responders did not participate in discovery efforts to identify risk loci for the GWAS [19]. Consequently, data from the WTC cohort serves as a valuable validation sample to test the out-of-sample prediction accuracy of a PRS for renal outcomes. Our primary hypothesis was that a PRS based on data from a large GWAS of rapid eGFR decline will be associated with early GFR decline, even after adjusting for traditional risk factors associated with progression of CKD. We also hypothesized that the PRS will be associated with individual differences in baseline eGFR, CKD stage, and eGFR trajectories.

## Methods

### Study design and setting

The WTC Health and Wellness Program is a prospective study that provides annual health monitoring visits for individuals who responded to the terrorist attacks that occurred on 9/11/2001. Information regarding patient recruitment and study procedures was published previously [33]. WTC responders for whom genotyping data and with three or more eGFR values were available were included in the study. The Stony Brook University Internal Review Board (IRB) approved the study procedures (IRB# 604,113). Measures of serum creatinine were obtained from blood samples collected between 2016 and 2020 during annual visits. eGFR was calculated using the CKD-EPI equation [34]. The observation period of the study was approximately 4 years for individual responders, beginning in 2016 and ending in 2020. (see Supplementary Fig. 1). No subjects in the study had end-stage kidney disease (ESKD). The analytic sample only included subjects that are predominantly European in ancestry, as determined by genetic principal components analysis (EUR > 0.80; *n* = 1,601), because the GWAS meta-analysis [19] from which the PRS was calculated included mostly participants with predominately European ancestry.

### Measurements

eGFR: The eGFR trajectories were estimated from 5,584 serum creatinine observations from a total of 1601 subjects using a linear mixed-effects model. This included three sequential yearly measures of obtained from 880 subjects, four measures from 661 subjects and five measures from another 60 participants. We defined the mean linear rate of eGFR change (in units of ml/min/1.73m^2^/year) as the total change in eGFR (rise or decline) from baseline to final value divided by the years of follow-up.

Covariates: Baseline demographic and health-associated factors known to be associated with a decline in eGFR were included in the analysis. These included self-reported demographic factors such as age [35], gender [36] and educational level [37]; as well as self-reported co-morbidities, including diabetes [6], hypertension [38], obesity (as determined by mean body mass index [BMI]) [39] and cardiovascular disease (defined by any of the following diagnosis: myocardial infarction, stroke, and heart failure) [40].

Finally, exposure severity at the time of the 9/11 terrorist attacks was included as a covariate due to the known association of air pollution with CKD [41]. The definition of the ‘exposure severity’ variable in the WTC cohort has been published previously [42] and consisted of four exposure groups: ‘very high’, ‘high’, ‘intermediate’, and ‘low’. These groups were defined as follows. ‘Very high’ exposure: those who worked more than 90 days at the WTC site, were exposed to the dust cloud of debris from the building collapse, and worked at least some time on the pile of debris. ‘High’ exposure: responders who were exposed to the dust cloud but either worked less than 90 days or did not work on the pile. ‘Intermediate’ exposure: responders who either worked between 40 days and 90 days or did not work on the pile and were not exposed to the dust cloud. ‘Low’ exposure: those who worked less than 40 days, did not work in the debris pile and were not exposed to dust from the collapse.

### eGFR outcomes

eGFR outcomes were operationalized based on the baseline renal function and its change during the observation period.

1. Continuous and Ordinal eGFR Outcomes:

- eGFR intercepts: estimated values of eGFR at the baseline assessment.
- CKD Stage: based on values of eGFR at the baseline assessment (Stage I ≥ 90, Stage II = 60–89, Stage III = 30–59, Stage IV = 15–29, Stage V = eGFR < 15, all values in ml/min/1.73 m [2]) per Kidney Disease: Improving Global Outcomes (KDIGO) guidelines [43].
- eGFR slopes: estimated rate of linear change in eGFR over the 3–5 year follow-up period. Annualized decline in eGFR was estimated as the mean of the random slope from the linear mixed effects model.

2. Longitudinal eGFR outcomes (Supplementary Fig. 2):

- Primary analysis: Results from previously published studies indicate that GFR typically declines by 0.8-1.0 ml/min/1.73 m [2] per year after the age of forty [44]. Therefore, we categorized GFR decline into two “clinical” categories (Supplementary Fig. 2 A): Subjects who exhibited ≥ 1.0 ml/min/1.73 m2/year decline in eGFR were categorized as “eGFR decline”, those with < 1.0 ml/min/1.73 m^2^/year decline in eGFR were categorized as “no eGFR decline” (reference group).
- Secondary analysis: we further divided subjects who exhibited a ≥ 1.0 ml/min/1.73 m^2^/year decline in eGFR into “mild” eGFR decline (i.e., 1.0 to 2.0 ml/min/1.73 m^2^/year) and “rapid” eGFR decline (> 2.0 ml/min/1.73 m^2^/year) (Supplementary Fig. 2B). These groups were compared to those who exhibited “no eGFR decline”. As was anticipated from the age and comorbidity distribution, we had only five subjects with a higher rate of eGFR decline (> 3.0 ml/min/1.73 m^2^/year) in our cohort.
- Sensitivity analysis: We noted a positive eGFR slope over time in a significant (~ 12%) proportion of subjects. Hence, “empirical” longitudinal eGFR categories were based on quartiles of eGFR slopes, with the lower-most quartile categorized as “eGFR decline” (Supplementary Fig. 2 C). The middle two quartiles were used as a reference group. Only 18 subjects had an eGFR rise of > 1.0 ml/min/1.73 m^2^/year (Supplementary Fig. 2D).

### Polygenic risk score (PRS) analysis

The PRS was constructed using PRSice 2.0 [45]. Variant effect sizes were based on the aforementioned GWAS of rapid GFR decline [19]. Further details of PRS analysis are mentioned in the Supplementary Methods section.

### Statistical methods

We tested the predictive validity of the PRS based on initial levels and linear rates of change in eGFR with all covariates indexed at the baseline assessment. A series of generalized linear models (GLMs) were estimated using the ‘MASS’ [46] and ‘nnet’ [47] packages in R. Covariates were added to each successive model to evaluate the strength and statistical significance of the PRS prediction after adjusting for population stratification, demographic factors, and comorbidities including body mass index [BMI] (measured in kg/m^2^), hypertension, diabetes and cardiovascular disease (self-reported). Multivariable linear regression was used to determine whether PRS predicted the eGFR intercepts and slopes. The mean of the slope was calculated using the mixed effects model for average rate of change in eGFR. Multivariable ordinal and multinomial logistic regression models were used to test whether the PRS predicted CKD stage and longitudinal eGFR categories, respectively, using the “no eGFR decline” or “middle quartiles” cohorts as the reference groups in multinomial models. Regression coefficients were reported for interval/ratio eGFR outcomes. Adjusted odds ratios (aORs) and risk ratios (aRRs) were reported for ordinal and categorical eGFR outcomes, respectively.

First, a series of univariate GLMs were estimated to assess the zero-order or unadjusted association between the PRS and eGFR outcomes. The first ten genetic principal components, age, biological sex, and education were then included as predictors of eGFR outcomes to adjust for potential confounding factors associated with population stratification and demographics. Next, BMI scores and dichotomous variables representing diabetes, hypertension, and cardiovascular disease (1 = Yes, 0 = No for each category) were included as additional predictors of eGFR outcomes to adjust for potential confounding factors based on individual differences in these established risk factors for CKD. Finally, a composite variable was introduced as a predictor of eGFR outcomes to capture variation in WTC exposure severity (“Low”, “Intermediate”, “High”, or “Very High”) [42]. To help facilitate the interpretation of estimated coefficients, the PRS was standardized (M = 0, SD = 1). Finally, we conducted two sensitivity analyses. First, given the small number of females (~ 7%, n = 141), we examined whether effects remained after excluding females from the sample (only males, n = 1485). Second, given heterogeneity in age, we restricted analyses to participants that were in the middle age range (45–65 years old) at the baseline assessment (*n* = 1239).

## Results

### Baseline characteristics

Our final analytic cohort included 1,601 subjects, predominately male (93%), with an average baseline age of 54.12 years [SD = 8.79; range from 35 to 84 years] (Table 1). Over 90% had a high school or college education. The calculated mean BMI was 30.96 (SD = 5.38) kg/m², with 50% obese. The prevalence of hypertension, diabetes, cardiovascular disease was 23%, 7% and 1% respectively. The mean baseline and final eGFR were 86.23 (SD = 13.82) and 83.57 (SD = 15.37) ml/min/1.73 m [2] respectively. The mean rate of eGFR decline was 0.75 (SD = 0.70) ml/min/1.73 m^2^/year. The prevalence of baseline CKD stages are reported in Supplementary Fig. 1. There were no patients with baseline CKD stages 4 and 5 in our cohort.

**Table 1:** Subject demographics, comorbidities and eGFR data

Age (years)	54.12	(8.18)
45–65 years old	1198	(75%)
Males	1485	(93%)
Female	116	(7%)
European Ancestry (EUR > 0.80)	1601	(100%)
Education		
No High School Diploma	42	(3%)
High School Diploma	297	(19%)
< Bachelor’s Degree	740	(46%)
Bachelors Degree or Graduate School	458	(29%)
Other or Unknown	64	(4%)
Body Mass Index (BMI, kg/m²)	30.96	(5.38)
Body Mass Index (BMI, kg/m²) > 30	801	(50%)
Hypertension	363	(23%)
Diabetes	106	(7%)
Cardiovascular Disease	18	(1%)
Baseline eGFR (ml/min/1.73 m²)	86.23	(13.82)
3rd eGFR measurement (ml/min/1.73 m²)	84.30	(14.32)
5th eGFR measurement (ml/min/1.73 m²)	83.57	(15.37)
Average Rate of GFR Decline (ml/min/1.73 m².per year)	0.75	(0.70)

**Notes.** Means are reported with standard deviations (SDs) in parentheses for variables measured on interval and ratio scales. Frequencies are reported with percentages in parentheses for variables measured on nominal and ordinal scales.

### Baseline characteristics of subjects in longitudinal eGFR categories

The sample characteristics for each category are reported in Tables 2 and 3. Based on the ‘clinical’ categorization, we determined that 553 subjects (34.5%) exhibited a decline in eGFR, while 1,048 exhibited a stable GFR (Table 2). For these groups, the mean rates of eGFR decline (SD) were 1.47 (0.43) and 0.38 (0.48) ml/min/1.73m^2^/year respectively. Based on the ‘empirical’ categorization, the mean rates of eGFR change (SD) were − 1.62 (0.41), + 0.11 (0.42) and − 0.75 (0.22) ml/min/1.73m^2^/year for the lower, upper and middle two quartiles respectively (Table 3).

**Table 2:** Categories Based on Clinical Cut-Off for Rates of Annual Change in eGFR

	No eGFR Decline (reference) (< 1.00 ml/min/1.73m^2^/year)		eGFR Decline (≥ 1.00 ml/min/1.73m^2^/year)		p-value
*n*	1048		553		
Age	53.68	(7.99)	54.95	(8.44)	.009^a^
Male	976	(93%)	509	(92%)	.486^b^
Education					.143^b^
No High School Diploma	33	(3%)	9	(2%)	
High School Diploma	187	(18%)	110	(20%)	
< Bachelor’s Degree	475	(45%)	265	(48%)	
BA/BS or Graduate Degree	307	(29%)	151	(27%)	
Other or Unknown	46	(3%)	18	(3%)	
BMI (kg/m²)	30.97	(5.54)	30.94	(5.06)	.468^a^
Hypertension	231	(23%)	132	(25%)	.404^b^
Diabetes	54	(5%)	52	(9%)	.001^c^
Cardiovascular Disease	8	(1%)	10	(2%)	.078^c^
Baseline eGFR (ml/min/1.73 m²)	86.89	(13.27)	84.97	(14.73)	.027^a^
3rd eGFR measurement (ml/min/1.73 m²)	89.03	(11.87)	75.32	(14.26)	< .001^a^
5th eGFR measurement (ml/min/1.73 m²)	89.67	(10.87)	72.24	(16.28)	< .001^a^
Average rate of eGFR decline (ml/min/1.73m^2^/year)	0.38	(0.48)	1.47	(0.43)	< .001^a^

**Notes.** Means are reported with SDs in parentheses for variables measured on interval and ratio scales. Frequencies are reported with percentages in parentheses for count variables measured on binary and ordinal scales. P-values for ^a^Kruskal-Wallis rank sum test, ^b^Pearson’s chi-squared test with Yate’s continuity correction, and ^c^Fisher’s exact test are reported.

**Table 3:** Empirical Categories Based on the Quartiles of Annual Change in eGFR

	Middle Quartiles (reference)	Lower Quartile	Upper Quartile	p-value
*n*	800	401	400	
Age	54.43 (7.69)	55.28 (8.73)	52.35 (8.25)	< 0.001^a^
Gender	740 (93%)	371 (93%)	374 (94%)	0.802^b^
Education				0.398^b^
No High School Diploma	20 (3%)	7 (2%)	15 (4%)	
High School Diploma	148 (18%)	82 (20%)	67 (17%)	
< Bachelor’s Degree	356 (44%)	192 (48%)	192 (48%)	
BA/BS or Graduate Degree	235 (29%)	110 (27%)	113 (28%)	
Other or Unknown	41 (5%)	10 (2)	13 (3%)	
BMI (kg/m²)	30.99 (5.59)	30.87 (4.88)	30.96 (5.43)	0.906^a^
Hypertension	186 (23%)	92 (23%)	85 (21%)	0.672^b^
Diabetes	44 (6%)	41 (10%)	21 (5%)	0.006^c^
Cardiovascular Disease	5 (< 1%)	9 (2%)	4 (1%)	0.050^c^
Baseline eGFR (ml/min/1.73 m²)	88.55 (13.20)	84.13 (15.21)	83.68 (12.77)	< 0.001^a^
3rd eGFR measurement (ml/min/1.73 m²)	86.15 (12.31)	73.15 (14.51)	91.76 (10.94)	< 0.001^a^
5th eGFR measurement (ml/min/1.73 m²)	80.47 (11.94)	70.43 (15.32)	94.32 (9.17)	< 0.001^a^
Average rate of eGFR change (ml/min/1.73m^2^/year)	-0.75 (0.22)	-1.62 (0.41)	+ 0.11 (0.42)	< 0.001^a^

**Notes.** Means are reported with standard deviations in parentheses for variables measured on interval and ratio scales, and frequencies are reported with percentages in parentheses for variables measured on nominal and ordinal scales. P-values for ^a^Kruskal-Wallis rank sum test, ^b^Pearson’s chi-squared test with Yate’s continuity correction, and ^c^Fisher’s exact test are reported.

Subjects exhibiting an eGFR decline were more likely to be diabetic compared to the reference group The baseline characteristics of the eGFR groups in our secondary analysis (“mild” and “rapid” eGFR decline *versus* “no eGFR decline”) are reported in Supplementary Table S1.

### Polygenic association of continuous and ordinal eGFR outcomes

The PRS for GFR decline was a significant predictor of eGFR at baseline, both before and after adjusting for covariates (Table 4, range of *b* = -0.77 to -1.01). Results were similar in sensitivity analyses after excluding females from the sample (*b* = -0.79, *CI.95%* = -1.40, -0.18) and including only participants 45–65 years old (*b* = -0.97, *CI.95% = -1.65, -0.29*). Age (*b* = -0.62, *CI.95% = -0.70, -0.56*), diabetes (b = -2.37, *CI.95% = -4.86, -0.31*), and cardiovascular disease (b = -5.72, CI.95% = -10.93, -0.46) were also associated with lower baseline GFR.

**Table 4:** Polygenic Associations of Continuous and Ordinal eGFR Outcomes

	Renal Outcome					
	**eGFR Intercept**		**eGFR Slope**		**CKD Stage**	
	*b* *(95% CI)*	*p*_*1*_ *p*_*2*_	*b* *(95% CI)*	*p*_*1*_ *p*_*2*_	*OR* *(95% CI)*	*p*_*1*_ *p*_*2*_
Zero-Order (not adjusted for covariates)	-0.77 (-1.36, -0.19)	0.010 0.010	-0.02 (-0.06, 0.01)	0.229 0.491	1.07 (1.01, 1.14)	0.021 0.021
Adjusted for age, gender, education, and the first ten genetic principal components	-1.01 (-1.57, -0.45)	< 0.001 0.002	-0.03 (-0.07, 0.00)	0.077 0.306	1.18 (1.06, 1.32)	0.003 0.008
+ adjusted for BMI, diabetes, hypertension, and cardiovascular disease	-1.00 (-1.57, -0.43)	< 0.001 0.002	-0.03 (-0.06, 0.01)	0.163 0.491	1.19 (1.07, 1.34)	0.002 0.008
+ adjusted for exposure severity	-1.00 (-1.59, -0.41)	< 0.001 0.002	-0.02 (-0.06, 0.02)	0.317 0.491	1.18 (1.05, 1.32)	0.007 0.014

**Notes.** Unstandardized regression coefficients (b) and odds ratios (OR) are reported for interval and ordinal outcomes, respectively, with 95% confidence intervals (CI) shown in parentheses just below each value. Polygenic risk scores (PRSs) were transformed to standardized scores (M = 0, SD = 1) to facilitate the interpretation of estimated coefficients. For example, for every incremental increase in SD in the PRS for rapid eGFR decline, the odds of having a *more* advanced stage of CKD (i.e., *Stage II* or *III* versus Stage I) increases by ~ 18%, holding all other variables constant. P-values indicate the probability of having observed the estimated association if the null hypotheses (b = 0 or OR = 1) were true, before (p_1_) and after (p_2_) adjustment for family-wise error using the Holm method, whereby each column of point estimates constitutes a “family” (i.e., four null hypothesis significance tests for each renal outcome).

The PRS was also significantly associated with more advanced baseline CKD stage both before and after adjusting for covariates (Table 4, range of OR = 1.07 to 1.18). Similar results were obtained after excluding females from the sample (aOR = 1.13, *CI.95%* = 1.00–1.28) and including only participants 45–65 years *(aOR = 1.19, CI.95% = 1.04–1.36)*. Age (*aOR* = 1.10, CI.95% = 1.08, 1.12), diabetes (*aOR* = 1.76, CI.95% = 1.06, 2.92), and cardiovascular disease *(aOR* = 4.45, CI.95% = 1.31, 15.07) were also associated with higher baseline CKD stage.

By contrast, the PRS for GFR decline was not significantly associated with eGFR slopes in the full sample (Table 4 (*b* = -0.02, *SE* = 0.01, *p* = 0.491)), among males (*b* = -0.03, *SE* = 0.02, *p* = 0.201) or participants 45–65 years old (*b* = -0.01, *SE* = 0.02, *p* = 0.676). As expected, older age at baseline was associated with a more rapid decline in eGFR (b = -0.01, SE = 0.002, p < 0.001).

### Polygenic association of longitudinal eGFR categories

The PRS was significantly associated with decline in eGFR, relative to no eGFR decline (as defined by ‘clinical’ categories), before and after adjusting for covariates (Table 5; *aOR* = 1.14, CI.95% = 1.01, 1.28),) in the overall cohort, after females were excluded from the sample (*aOR* = 1.13, CI.95% = 1.00, 1.28), and for participants 45–65 years old (aOR = 1.15, CI.95% = 1.01, 1.33). Diabetes was associated with a higher risk of eGFR decline (aOR = 1.71, CI.95% = 1.13, 2.60). Our secondary analysis revealed that the PRS was even more strongly associated with a “rapid” decline in eGFR compared to those exhibiting no eGFR decline (Supplementary Table S2). The PRS was also significantly associated with the lower quartile of eGFR change over time, relative to the middle quartiles (fully adjusted *OR* = 1.20; CI.95% = 1.04, 1.38; Table 6), and not significantly associated with the upper eGFR quartile (all p-values > 0.05). The association between the lower quartile of eGFR change and the PRS decreased after excluding females from the sample (*aOR* = 1.15, *CI.95%* = 1.00–1.33) and remained unchanged for participants 45–65 years old (*aOR* = 1.22, *CI.95%* = 1.04–1.43). Older age was significantly associated with a lower risk of being in the upper quartile (*aRR* = 0.97, CI.95% = 0.95, 0.98), and cardiovascular disease with higher risk being in the lower quartile (*aRR* = 4.83, CI.95% = 1.43, 16.30) relative to the middle quartiles.

**Table 5:** Polygenic Associations of eGFR Decline

	Clinical eGFR Categories		
	**No eGFR Decline**	**eGFR Decline** **(** **≤** **-1.00 ml/min/1.73m** ^**2**^ **/year)**	
		*OR* *(95% CI)*	*p*_*1*_ *p*_*2*_
Zero-Order (not adjusted for covariates)	1.00	1.14 (1.03, 1.27)	0.011 0.034
Adjusted for age, gender, education, and the first ten genetic principal components	1.00	1.17 (1.04, 1.31)	0.008 0.031
+ adjusted for BMI, diabetes, hypertension, and cardiovascular disease	1.00	1.15 (1.03, 1.29)	0.016 0.034
+ adjusted for exposure severity	1.00	1.14 (1.01, 1.28)	0.036 0.036

**Notes.** Decline = ≤ -1.00 ml/min/1.73m^2^/year. No Decline = > -1.00 ml/min/1.73m^2^/year. Odds ratios (OR) are reported with 95% CI in parentheses just below point estimates. PRSs were transformed to standardized scores (M = 0, SD = 1) to facilitate the interpretation of estimated coefficients. For example, for every one-point increase in the SD of the PRS, the odds of eGFR decline, compared to no GFR decline, increases by 14%, when holding all other variables constant. P-values indicate the probability of having observed the estimated association if the null hypothesis (no association; OR = 1) were true, before (p_1_) and after (p_2_) adjustment for family-wise error using the Holm method, whereby the reported column of point estimates constitutes a “family”.

**Table 6:** Polygenic Associations of Upper and Lower Quartiles representing Linear Changes in eGFR

	Empirical eGFR Categories				
	**Middle Quartiles**	**Lower Quartile**		**Upper Quartile**	
		*RR* *(95% CI)*	*p*_*1*_ *p*_*2*_	*RR* *(95% CI)*	*p*_*1*_ *p*_*2*_
Zero-Order Association (not adjusted for covariates)	1.00	1.18 (1.05, 1.33)	0.007 0.014	1.04 (0.92, 1.17)	0.541 1.000
Adjusted for age, gender, education, and the first ten genetic principal components	1.00	1.22 (1.07, 1.38)	0.003 0.009	1.01 (0.89, 1.15)	0.821 1.000
+ adjusted for BMI, diabetes, hypertension, and cardiovascular disease	1.00	1.21 (1.06, 1.38)	0.004 0.012	1.04 (0.91, 1.18)	0.565 1.000
+ adjusted for exposure severity	1.00	1.20 (1.04, 1.38)	0.010 0.014	1.07 (0.93, 1.23)	0.343 1.000

**Notes.** Unadjusted and adjusted relative risk ratios (RR) are reported with 95% confidence intervals (CI) in parentheses. The p-values indicate the probability of having observed the estimated association if the null hypotheses of no association (RR = 1.00) were true, unadjusted for family-wise error rate. P-values adjusted for family-wise error rate using the Holm method are reported just below unadjusted p-values.

## Discussion

Little is known about the predictors and correlates of eGFR decline in adults with a low CKD risk based on traditional risk factors. In this study, we evaluated the utility of polygenic risk scores (PRS) to predict renal outcomes including declining eGFR at midlife. To the best of our knowledge, this is the first clinical translation study to examine the use of a PRS constructed from a large GWAS of rapid eGFR decline [19] for renal outcomes in an independent longitudinal cohort of adults. We found that our calculated PRS was significantly associated with several renal outcomes, including baseline eGFR, higher CKD stage and eGFR decline (relative to those exhibiting a stable eGFR over time) after adjusting for traditional risk factors.

The present study extends the body of work on the use of PRS to determine the risk for kidney disease^23,28−31^. Unlike previous studies that used selected gene variants, we constructed and validated a PRS using a p-value threshold of 1.00 that permitted us to identify the aggregate effects of over 400,000 SNPs. Our study is also unique by focusing on the largest and most recent GWAS to date [19], that was specifically designed to evaluate rapidly declining eGFR (unlike previous GWASs that focused on a one-time eGFR measure or incident CKD). The GWAS meta-analysis reported by Gorski et al. [19] included a very large sample (> 270,000 individuals) with at least two assessments of kidney function. Exaggerated GFR decline over time (beyond that expected with advancing age) is known significantly to increase the long-term risk of developing ESKD [2]. Identification of individuals at high risk for ESKD at a younger age and early in the course of their disease can lead to ‘targeted’ clinical studies and the development of preventive therapies that are more likely to be both high yield and cost-effective.

We also used the PRS to evaluate cases of longitudinal rise in eGFR. An abnormal rise in GFR is sometimes noted early in pathogenesis of some kidney diseases e.g. diabetic nephropathy [48]. This observation is consistent with ‘glomerular hyperfiltration’, which is a condition known to be associated with cardiovascular disease and morality [49–51]. 50% of subjects in our cohort were obese and obesity has been associated with the development of CKD possibly via direct effects on compensatory glomerular hyperfiltration [52, 53]. However, the PRS calculated for a rapid decline in eGFR was not associated with a rise in eGFR over time either before or after adjustment for BMI or diabetes (upper quartile; Table 6). To the extent that underlying genetic factors contribute to increased liability for glomerular hyperfiltration, these findings suggest that the relevant variants differ from those that contribute to decline in eGFR.

Other strengths of this study include the use of a large cohort of relatively young and healthy adults without existing CKD with serial eGFR measures. The average baseline age of our cohort was ~ 49.7 years, while most studies in the Gorski et meta-analysis included subjects with baseline age > 60 years [19]. The prevalence of hypertension, diabetes, cardiovascular disease was 23%, 7% and 1% respectively; was significantly lower than the overall prevalence in the United States, estimated at 47%, 10.5% and 6.7% respectively. [54] This permitted us to evaluate the genomic basis of early decline GFR before the pathogenic effects of older age and chronic medical conditions set in. We conducted a secondary analysis of those with more “rapid” GFR decline (> 2.0 ml/min/1.73m^2^/year) that provided a proof of concept; with a stronger association of the PRS observed with more severe eGFR decline (fully adjusted OR 1.14 vs. 1.37). We obtained consistent results in our sensitivity analysis using “empirical” categories of GFR change over time. In addition to our longitudinal evaluation, we also used our calculated PRS to predict other renal phenotypes including baseline eGFR, eGFR slope and CKD stage. The eGFR measurements reported for subjects in the WTC cohort were performed in a health monitoring visit (i.e., ‘steady-state’) setting. Thus, we avoided the problems typically associated with studies based on EHR data that could include eGFR measurements performed during hospitalizations often in the setting of acute kidney injury. Recent data suggests an association of air pollution with toxins, a significant issue noted in 9/11 first responders, with CKD [55]. Our data shows a polygenic association with renal outcomes even after adjusting for exposure severity. This study adds to the research on the long-term health consequences suffered by WTC first responders. It also provides a stimulus for further research on the genomics of early kidney disease, as our findings focus on young and middle-aged adults who maybe at high risk and thus more likely to benefit from critical preventive therapies.

Our study also has significant limitations. This is an observational study that cannot establish causality, although SNPs for each individual person are fixed at the time of gamete formation before changes in eGFR over time thereby establishing temporal precedence. We note the association of PRS with baseline eGFR and eGFR-based higher CKD stage, which are both ‘static’ measures that were ‘fixed’ at the start of our observation period. One can postulate that the subjects identified with an increased genetic risk might already be vulnerable to GFR decline since childhood. Studies that measure serial eGFRs from infancy to adulthood will be required to test this hypothesis. While, the size of estimated effects was somewhat modest, with relative risks ranging from 1.14 to 1.42, they are similar to the effect sizes reported in previous PRSs for renal outcomes [23, 28−31]. Our study also revealed no association between the PRS and linear rates of eGFR change in the overall cohort. This observation may be due to the relatively short follow-up time (~ three to five years).

While the serum creatinine based GFR estimating equations are commonly used to track kidney function in clinical practice, serum creatinine levels are influenced by non-GFR determinants, e.g., diet and muscle mass. While we controlled for BMI in our adjusted models and restricted analyses to participants with predominately European ancestry, there is a possibility that the PRS might influence one or more of the non-GFR-determinants of serum creatinine. Another limitation is that we did not have data on proteinuria, a variable that is known to be associated with the progression of CKD [56]. The subjects in the WTC cohort were relatively young and were diagnosed with few co-morbid conditions. Thus, we could not determine the association of our PRS with the ‘hard’ renal outcomes e.g., doubling of serum creatinine levels or ESKD. Our findings are also limited by the fact that the co-morbid conditions were all self-reported and were not defined via diagnostic codes and data on the duration of comorbidities was not available. Our study was restricted to predominantly Americans of European ancestry. Studies involving genomic data to calculate PRSs from other ancestries are required to reduce health care disparities. Recent data suggests that women compared to men have lower decline in GFR over time [57]. While our sensitivity analysis restricted to only men showed consistent results, our cohort had very few females (7%). Future studies to the genetic risk of sex-related differences in GFR decline and CKD are warranted. Finally, most subjects were first responders (police officers, EMT, construction workers). These factors limit the generalizability of our findings.

In conclusion, we report that genetic markers in the aggregate are associated with decline in eGFR among middle-aged, relatively healthy individuals even after accounting for traditional risk factors of CKD progression. Our findings will need to be validated in larger multi-ethnic cohorts with longer follow-up periods to provide insight into the potential associations with advanced kidney disease.

## Electronic supplementary material

Below is the link to the electronic supplementary material.

Supplementary Material 1

## Data Availability

The datasets used and/or analyzed during the current study are available from the corresponding author on reasonable request. Dataset are available in the following repository: https://osf.io/x5cwy/files/. Title of the repository: FK_WTC_PRS.

## Declarations

### Experiments on humans and/or the use of human tissue

All methods were carried out in accordance with relevant guidelines and regulations. All experimental protocols were approved by the Stony Brook University Internal Review Board (IRB) approved the study procedures (IRB# 604113). Informed consent was obtained from all subjects and/or their legal guardian.
