# Supplementary material for: Polygenic association of glomerular filtration rate decline in world trade center responders
Source: BMC Nephrol. 2022 Oct 28;23:347. doi: 10.1186/s12882-022-02967-5 (PMC9615399; doi:10.1186/s12882-022-02967-5)
Supplement: Supplementary file 1 — Supplementary Material 1 [file 12882_2022_2967_MOESM1_ESM.docx]

**SUPPLEMENTARY SECTION:**

**Manuscript Title: Polygenic Association of Glomerular Filtration Rate Decline in World Trade Center Responders**

**Authors:** Farrukh M. Koraishy, Frank D. Mann, Monika A. Waszczuk, Pei-Fen Kuan, Katherine Jonas, Xiaohua Yang, Anna Docherty, Andrey Shabalin, Cassianne Robinson-Cohen, Sean Clouston, Roman Kotov, and Benjamin Luft

**Index:**

1. Supplementary Methods
2. Supplementary Table 1 & 2
3. Supplementary Figures 1 & 2

**Supplementary Methods:**

**CKD EPI equation:**

The CKD-EPI equation, expressed as a single equation, is GFR = 141 × min(Scr/κ, 1)α × max(Scr/κ, 1)-1.209 × 0.993Age × 1.018 [if female] _ 1.159 [if black], where Scr is serum creatinine, κ is 0.7 for females and 0.9 for males, α is -0.329 for females and -0.411 for males, min indicates the minimum of Scr/κor 1, and max indicates the maximum of Scr/κ or 1.

**Brief description of the Gorki et al meta-analysis:**

This is was a meta-analysis of 42 GWAS studies from the Chronic Kidney Diseases Genetics Consortium and United Kingdom Biobank to identify genetic loci for rapid eGFRcrea decline.

Definitions of eGFRcrea decline used:

1. “Rapid3”: 3 mL/min/1.73m2/year or more. This included 34,874cases and 107,090 controls

2. “CKDi25”: eGFRcrea decline 25% or more and eGFRcrea under 60 mL/min/1.73m2 at follow-up among those with eGFRcrea 60 mL/min/1.73m2 or more at baseline. This included 19,901 cases and 175,244 controls.

Since our cohort included only five subjects with a rate of eGFR decline of >3 ml/min/1.73 m2 per year, we focused on testing the association of PRS for rapid eGFR decline based on the aforementioned “CKDi25” criteria.

Key results: Seven independent variants were identified across six loci for Rapid3 and/or CKDi25. Five lead variants at four loci with genome-wide significance (near UMOD-PDILT (two), PRKAG2, WDR72, OR2S2) and two variants among 265 known eGFRcrea variants (near GATM, LARP4B).

The summary description of the study population of each of the 42 GWAS studies in this meta-analysis is described in Supplementary Table 1 in the Gorki et al manuscript (available online). Briefly, of the 42 studies, 34 were European ancestry, while 2 were African American, 2 South Asian and 4 studies had mixed ancestry. The distribution of male gender was 30-80% and age range was 33 to 72 years. Baseline eGFR ranged from 49.4 to 111.5 ml/min/ 1.73m2 and follow-up time ranged from 1.00 to 15.14 years.

**Data Analytic Procedures:**

Individual trajectory plots of eGFR for each subject were then plotted against assessment dates (Panel A, Supplementary Figure 1). Box and whisker plots of eGFR were plotted describing the mean eGFR at each assessment (Panel B, Supplementary Figure 1). Finally, bar plots of eGFR were plotted describing the percentage of subjects diagnosed with eGFR-based CKD stages I-V at each assessment (Panel C, Supplementary Figure 1). These plots were visually inspected before estimating random intercepts and slopes to operationalize eGFR outcomes. Briefly, a repeated-measures mixed-effects model was fitted to the data using a restricted maximum likelihood approach as in the ‘lme4’ and ‘nlme’ packages in R, written as:${eGFR}_{ti}=\left( \beta_{1}+d_{1i} \right)+(\beta_{2}+d_{2i})\times\frac{t-k_{1}}{k_{2}}+u_{ti}$, where ${eGFR}_{ti}$ is eGFR measured at time *t* for subject *i*.^1^ The random intercept for subject *i* $\left( \beta_{1}+d_{1i} \right)$ is equal to the sum of the sample mean ($\beta_{1})$and the subject’s individual deviation from that mean$\left( d_{1i} \right)$. The random slope for subject *i*$(\beta_{2}+d_{2i})$ *i*s the sum of the sample mean of the slope ($\beta_{2})$ and the subject’s individual deviation from the sample mean of the slope${(d}_{2i})$, and $u_{ti}$ is the measurement-specific error term (i.e. residual) at time *t* for subject *i*. Finally, $k_{1}=1$ centers the intercept at baseline, while $k_{2}=1$ scales the slope in original units of time in order to represent the predicted linear change in eGFR for subject *i* over the course of one year^1^.

Using Satterthwaite's method, we found that the predicted average rate of change in eGFR was negative and significantly different from zero ($\beta_{2}$= -0.75, *SE* = 0.08, *t* = -9.24, *p* < .001), as was the mean intercept ($\beta_{1}$= 86.17, standard error *[SE]* = 0.34, *t* = 254.96, *p* < .001). The mixed effects model explained a substantial portion of the total variance in eGFR (conditional R^2^ = 0.820). A relatively small portion of the variance was accounted for by fixed effects alone (marginal *R^2^* = .004), which reflected a high degree of variability among the eGFR trajectories. Random intercepts and slopes were then presented as new variables used to capture both initial levels and rates of change in eGFR and to operationalize renal outcomes for PRS analyses.

**Polygenic Risk Score (PRS) Analysis:**

Formula for the polygenic risk score (PRS):($\mathrm{PRS}_{k}= \sum_{i} \beta_{i}\mathrm{SNP}_{ik}$, whereby _i_ is the ith SNP of all SNPs associated with variable *k,* and $\beta_{i}$is the effect size of the ith SNP in the discovery GWAS). The PRS was calculated using the aggregate effects of over 400,000 SNPs. Please refer to the original study by Gorski et al^2^ for the complete list of the SNPs that were used.

Our analysis used a full list of SNPs after clumping and their corresponding weights from the GWAS discovery sample (p-value threshold = 1). Notably, Gorski et al^2^, used two different definitions of “rapid” decline in GFR. “Rapid3” cases were those with an eGFRcrea decline of >3.0 ml/min/1.73 m^2^ per year compared to those with “no decline” (-1 to +1 ml/min/1.73 m^2^ per year). By contrast, “CKDi25” cases were those with a ≥25% decline in eGFRcrea plus change from a baseline eGFRcrea ≥60 to eGFRcrea <60 ml/min/1.73 m^2^ at follow-up, while “CKDi25” controls maintained an eGFRcrea ≥60 ml/min/1.73 m^2^ at baseline and follow-up. Since our cohort included only five subjects with a rate of eGFR decline of >3 ml/min/1.73 m^2^ per year, we focused on testing the association of PRS for rapid eGFR decline based on the aforementioned “CKDi25” criteria.

DNA extracted from peripheral whole blood mononuclear cells obtained from the WTC responders was genotyped using the Infinium Global Screening Array (Illumina, San Diego, CA, USA). SNPs were identified and processed via established protocols for genotype calling, filtering, ancestry measurement and imputation. Genotypes were imputed to 25,514,638 million SNPs using the Haplotype Reference Consortium reference panel on the Michigan Imputation Server pipeline v1.2.4^3^.

Before imputation, the genotypes were filtered for ambiguous strand orientation, missingness rate>5% (by marker exclusion, then by individual), Hardy-Weinberg equilibrium violation (p<10-6), sex mismatch (“sex check” function for X chromosome homozygosity estimate), and non-European ancestry (principal component analysis against the reference panel from the 1000 Genomes data). After imputation, SNPs were excluded for imputation R2<0.5, average call rate below 90% and minor allele frequency (MAF) below 0.1%. PLINK was used to handle genetic data and perform quality control^4^. The pi_hat>.125 was used for the identity by descent exclusion (one sample per pair was removed at random), and samples of less than 80% genetic European ancestry were excluded.

| **Supplementary Table S1. Clinical Categories Based on Mild eGFR Decline and Rapid eGFR Decline *versus* No eGFR Decline** | | | | | | | |
| --- | --- | --- | --- | --- | --- | --- | --- |
|  | **No eGFR Decline (reference)**  **(<1.0ml/min/1.73m^2^/year)** | | **Mild eGFR decline**  **(1.0 - 2.0 ml/min/1.73m^2^/year)** | | **Rapid eGFR decline**  **(>2.0 ml/min/1.73m^2^/year)** | | **p-value** |
| n | 1048 | | 497 | | 56 | |  |
| Age | 53.68 | (7.99) | 54.68 | (8.26) | 57.31 | (10.01) | 0.003^a^ |
| Male | 976 | (93%) | 457 | (92%) | 52 | (93%) | 0.679^c^ |
| Education |  |  |  |  |  |  | 0.347^b^ |
| No High School Diploma | 33 | (3%) | 7 | (1%) | 2 | (4%) |  |
| High School Diploma | 187 | (18%) | 99 | (20%) | 11 | (30%) |  |
| < Bachelor’s Degree | 475 | (45%) | 242 | (49%) | 23 | (41%) |  |
| BA/BS or Graduate Degree | 307 | (29%) | 133 | (27%) | 18 | (32%) |  |
| Other or Unknown | 46 | (3%) | 16 | (1%) | 2 | (4%) |  |
| Body Mass Index | 30.97 | (5.54) | 30.92 | (5.07) | 31.15 | (5.01) | 0.692^a^ |
| Hypertension | 231 | (22%) | 111 | (23%) | 21 | (39%) | 0.022^b^ |
| Diabetes | 54 | (5%) | 43 | (9%) | 9 | (16%) | < 0.001^c^ |
| Cardiovascular Disease | 8 | (< 1%) | 9 | (2%) | 1 | (2%) | 0.113^c^ |
| Baseline eGFR | 86.89 | (13.27) | 85.08 | (14.39) | 83.98 | (17.62) | 0.073^a^ |
| 3^rd^ eGFR | 89.03 | (11.87) | 76.33 | (13.50) | 66.30 | (17.44) | < 0.001^a^ |
| 5^th^ eGFR | 89.66 | (10.87) | 71.55 | (16.44) | 76.33 | (17.95) | < 0.001^a^ |
| Rate of eGFR decline  (ml/min/1.73m^2^/year) | 0.37 | (0.48) | 1.36 | (0.27) | 2.42 | (0.43) | < 0.001^a^ |
| **Notes.** Means are reported with standard deviations (SDs) in parentheses for variables measured on interval and ratio scales. Frequencies are reported with percentages in parentheses for count variables measured on nominal and ordinal scales. P-values for ^a^Kruskal-Wallis rank sum test, ^b^Pearson’s chi-squared test with Yate’s continuity correction, and ^c^Fisher’s exact test are reported. | | | | | | | |

| **Supplementary Table S2. Polygenic Associations of Mild and Rapid eGFR Decline** | | | | | |
| --- | --- | --- | --- | --- | --- |
|  | **Clinical GFR Categories** | | | | |
|  | **No Decline** | **Mild GFR decline**  **(>1.0 -2.0 ml/min/1.73m^2^/year)** | | **Rapid GFR decline**  **(>2.0 ml/min/1.73m^2^/year)** | |
|  |  | *RR*  *(95% CI)* | *p_1_*  *p_2_* | *RR*  *(95% CI)* | *p_1_*  *p_2_* |
| Zero-Order Association  (not adjusted for covariates) | 1.00 | 1.14  (1.02, 1.27) | 0.017  0.069 | 1.18  (0.90, 1.54) | 0.232  0.232 |
| Adjusted for age, gender, education, and the first ten genetic principal components | 1.00 | 1.15  (1.02, 1.29) | 0.016  0.069 | 1.39  (1.05, 1.83) | 0.020  0.061 |
| + adjusted for BMI, diabetes, hypertension, and cardiovascular disease | 1.00 | 1.13  (1.01, 1.27) | 0.041  0.083 | 1.42  (1.07, 1.88) | 0.014  0.056 |
| + adjusted for exposure severity | 1.00 | 1.12  (0.99, 1.27) | 0.067  0.083 | 1.36  (0.99, 1.86) | 0.055  0.111 |
| **Notes.** Risk ratios (RR) are reported with 95% confidence intervals (CI) in parentheses. P-values indicate the probability of having observed the estimated association if the null hypotheses (no association; RR = 1.00) were true, unadjusted for family-wise error rate. P-values adjusted for family-wise error rate using the Holm method are reported just below unadjusted p-values. | | | | | |

**Supplementary Figures and Legends:**

**Supplementary Figure 1. Individual trajectory, box and whisker, and bar plots of eGFR**


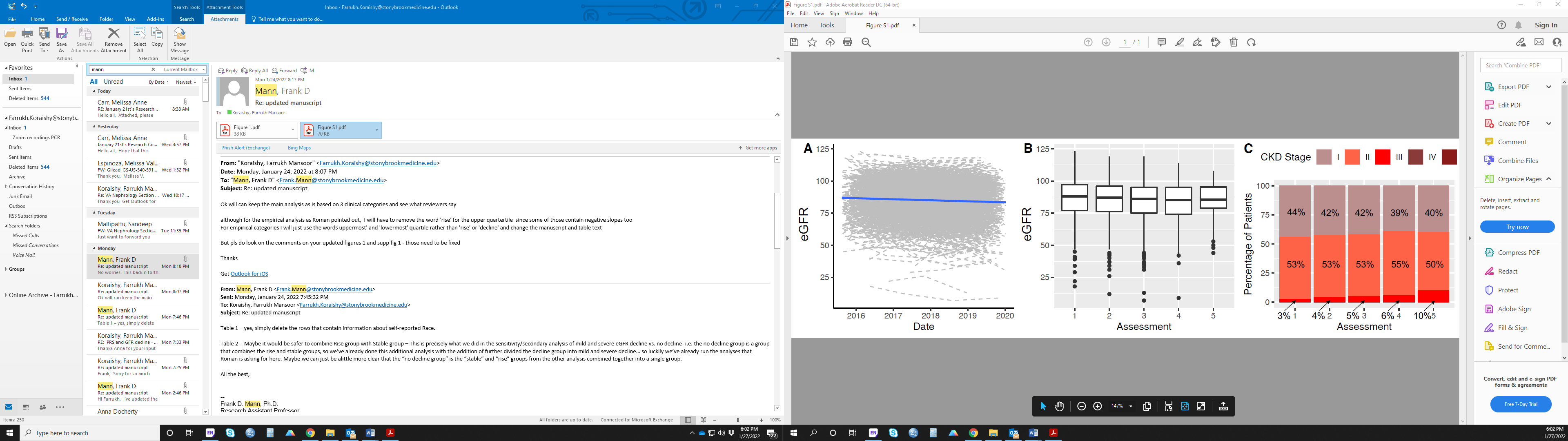


**Supplementary Figure 1. Individual Trajectory, Box and Whisker, and Bar Plots of eGFR.**

**A.** Individual eGFR trajectories across three or more annual assessments are shown by the gray dashed lines. The mean eGFR trajectory is indicated by the solid blue line. **B.** Box and whisker plots documenting the first quartile, median, and third quartile of eGFRs for study participants at the first, second, third, fourth, and fifth assessment. **C.** Bar plot depicting the percentage of patients at each assessment with various CKD stages based on eGFR. There were no patients with CKD stages 4 and 5.

**Supplementary Figure 2**

**Supplementary Figure 2. Frequency distributions of eGFR slopes from linear mixed effects model divided into longitudinal eGFR categories.** A., B., and D. are distribution of study subjects in eGFR categories based on clinical categories. C. Distributions of study subjects based on empirical categories

1. Grimm KJ, Ram, N., & Estabrook, R. . *Growth modeling: Structural equation and multilevel modeling approaches. Guilford Publications.*: The Guilford Press; 2016.

2. Gorski M, Jung B, Li Y, et al. Meta-analysis uncovers genome-wide significant variants for rapid kidney function decline. *Kidney international.* 2021;99(4):926-939.

3. Christian Fuchsberger LF, Sebastian Schoenherr, Sayantan Das, Gonçalo Abecasis. Michigan Imputation Server. <https://imputationserver.sph.umich.edu/index.html#>! Published 2021. Accessed2021.

4. Purcell S, Neale B, Todd-Brown K, et al. PLINK: a tool set for whole-genome association and population-based linkage analyses. *American journal of human genetics.* 2007;81(3):559-575.
